# The Prognostic Significance of KIAA1522 Expression in Non-Small-Cell Lung Cancer Patients

**DOI:** 10.7759/cureus.44016

**Published:** 2023-08-24

**Authors:** Murat Özdede, Hakan Taban, Orkun Akman, Sevgen Önder, Saadettin Kılıçkap

**Affiliations:** 1 Internal Medicine, Hacettepe University Faculty of Medicine, Ankara, TUR; 2 Medical Oncology, Hacettepe University Faculty of Medicine, Ankara, TUR; 3 Pathology, Hacettepe University Faculty of Medicine, Ankara, TUR

**Keywords:** survival outcomes, immunohistochemistry (ihc), kiaa1522, non-small cell lung cancer (nsclc), lung cancer

## Abstract

The majority of lung cancers belong to the non-small-cell lung cancer (NSCLC) category, which is linked to a high mortality rate despite significant progress in diagnosis and treatment. Therefore, there is a need for novel prognostic NSCLC biomarkers to improve prognosis which currently remains poor. Recent studies and analyses of gene expression data of NSCLC revealed that high expression of KIAA1522 was significantly associated with poor prognosis and decreased overall survival.

We identified 98 patients who underwent radical curative surgical resections or metastasectomy for pulmonary adenocarcinoma and squamous cell carcinoma at our institution or the pathological diagnosis confirmed by our pathologists. Following the latest data, we utilized immunohistochemistry to assess the expression of KIAA1522 and investigated its association with various clinic-demographic parameters, pathological stages, recurrence rates, overall survival, and disease-free survival in patients who achieved complete remission. Notably, there were no significant differences in the expression profiles of KIAA1522 between adenocarcinoma and squamous cell carcinoma samples (p=0.6). Survival analysis was conducted using log-rank tests and a multivariate Cox proportional hazard model.

Of the 98 samples, 54 (55.1%) exhibited high expression of KIAA1522, and patients with high KIAA1522 expression had a significantly shorter overall survival than the low-expression group (p=0.01). Multivariate Cox proportional hazard models in which metastatic patients were included revealed that along with older age, higher TNM stage (tumor, node, metastasis system), and Eastern Cooperative Oncology Group (ECOG) performance status, high expression of KIAA1522 served as an independent prognostic factor. A high expression profile was not significantly associated with relapses in those whose complete remission had been achieved. Still, those patients with high expression of KIAA1522 tended to exhibit a shorter disease-free survival rate.

In conclusion, our findings suggest that KIAA1522 expression is an independent factor for predicting overall survival and may serve as a valuable prognostic indicator for relapse and disease-free survival in NSCLC patients.

## Introduction

Lung cancer is a widespread malignancy causing the most cancer-related deaths globally [1,2]. Non-small-cell lung cancer (NSCLC), comprising over 85% of lung cancer cases, is notably diverse, leading to varied prognostic implications requiring distinct strategies [1,3,4]. Despite progress in prognostic categorization and personalized advanced therapies, the prognosis for NSCLC patients, especially those with local aggressive or metastatic disease, remains notably poor [5].

Novel biomarkers offer enhanced risk assessment and individualization in patient care. Aiming to uncover genes contributing to tumorigenesis, exploration of the gene expression data of NSCLC from Gene Expression Omnibus (GEO) revealed KIAA1522's association with poor prognosis. KIAA1522, a phosphoprotein with unclear functions but linked to RAS activity, shows high expression across various cancers [6,7]. Notably, it is linked to poor survival in colorectal, hepatocellular, and esophageal cancers [7-10].

Three significant studies explore the function of KIAA1522 in NSCLC. The initial research proposes KIAA1522 as a potential early lung cancer biomarker in bronchial brushing specimens [11]. The second study, carried out by the same group, delves deeper into the protein's role, unveiling its capacity to forecast prognosis and associated oncologic pathways [7]. The most recent study highlights a link between aberrant expression of KIAA1522 and resistance to platinum-based chemotherapy [12].

Our study followed the footsteps of previous works and analyzed the GSE32863 and GSE19188 datasets by BRB Array Tool, and confirmed the potential role of KIAA1522, as the transcript levels were significantly higher in tumor tissue than adjacent non-tumor tissues in both datasets [13,14]. Our study extends findings by evaluating KIAA1522's role through NSCLC samples via immunohistochemistry (IHC), assessing survival rates and relapse-progression, and constructing hazard models incorporating clinicopathological features and KIAA1522 expression patterns.

## Materials and methods

Clinical data

**Figure 1:**
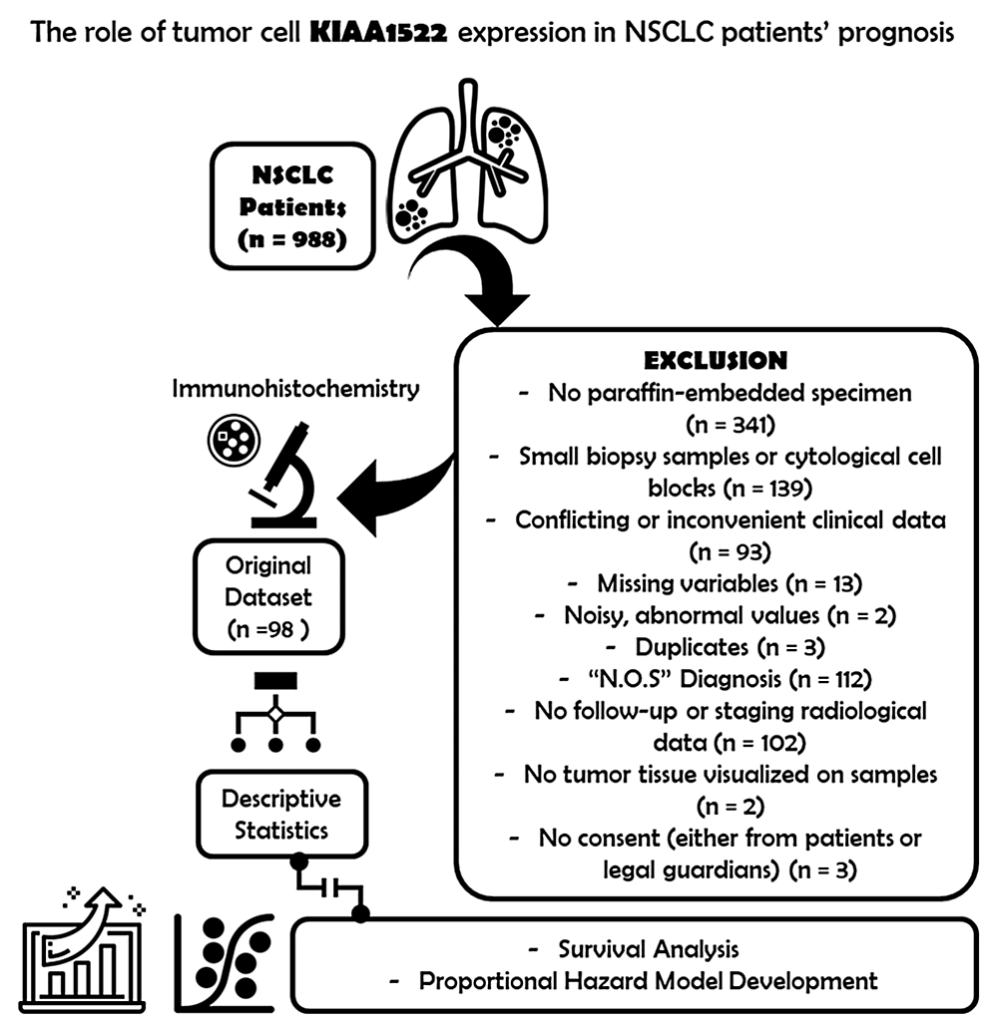
Study flowchart Patient inclusion process by review of 988 patients who underwent diagnostic and therapeutic procedures for lung cancer during the five-year period from 2010 to 2015. NSCLC, non-small-cell lung cancer.

We conducted a retrospective analysis of 98 Caucasian patients with NSCLC who underwent surgical tumor resection and whose tissue samples were evaluated and diagnosed at our institution between 2010 and 2015 (Figure 1). Patients under 18 years of age, those with insufficient tissue material or unavailable paraffin-embedded tissue specimens, incomplete radiological or medical data, and those diagnosed with unclassified, bronchial gland, neuroendocrine, large cell, carcinoid, neuroendocrine tumors, and metastatic lung diseases were excluded from the study. We also excluded cases of recurrent lung cancer, synchronous cancer, or a history of another primary tumor before the NSCLC diagnosis (except for cases of non-melanomatous skin and papillary thyroid cancer in which remission had been achieved).

Comprehensive electronic medical records review provided clinical and pathological data, including age, sex, comorbidities, drug history, smoking status, and ECOG performance. Dates of diagnosis confirmation, surgery, treatment, adverse effects, and disease progression were recorded. Charlson-comorbidity scores transformed comorbidity data. The assessment of disease response was made using Response Evaluation Criteria In Solid Tumors (RECIST). Progression-free survival (PFS) measured treatment start to last visit without progression/progression/death, while overall survival (OS) measured treatment start to death/last visit. Median survival was calculated for groups with fewer than half surviving.

Informed consent was obtained from each living patient and from the first-degree relatives of deceased patients included in the study. All human subjects or their legal guardians were informed that surplus tissue samples and associated data would be used solely for research purposes. Three patients who survived opted out of sharing future survival data by not attending follow-ups after enrollment. Consequently, their last visits were censored, and no data were collected or analyzed beyond that point to honor their preferences. Data were also censored for patients surviving beyond five years post-diagnosis.

IHC data (TTF-1, CK, p63, etc.) and molecular studies (ALK/ROS-1, EGFR, specific mutations, etc.) were collected from the institutional database. Radiological views and reports were also sourced from this database, with staging updated to the 8th TNM system edition (American Joint Committee on Cancer) for cases initially reported under the 7th edition. Patient confidentiality was upheld using unique five-digit codes. The university's non-interventional clinical research ethics committee approved the study (GO 16/761-26, date: 06.12.2016).

Tissue samples and IHC

**Figure 2:**
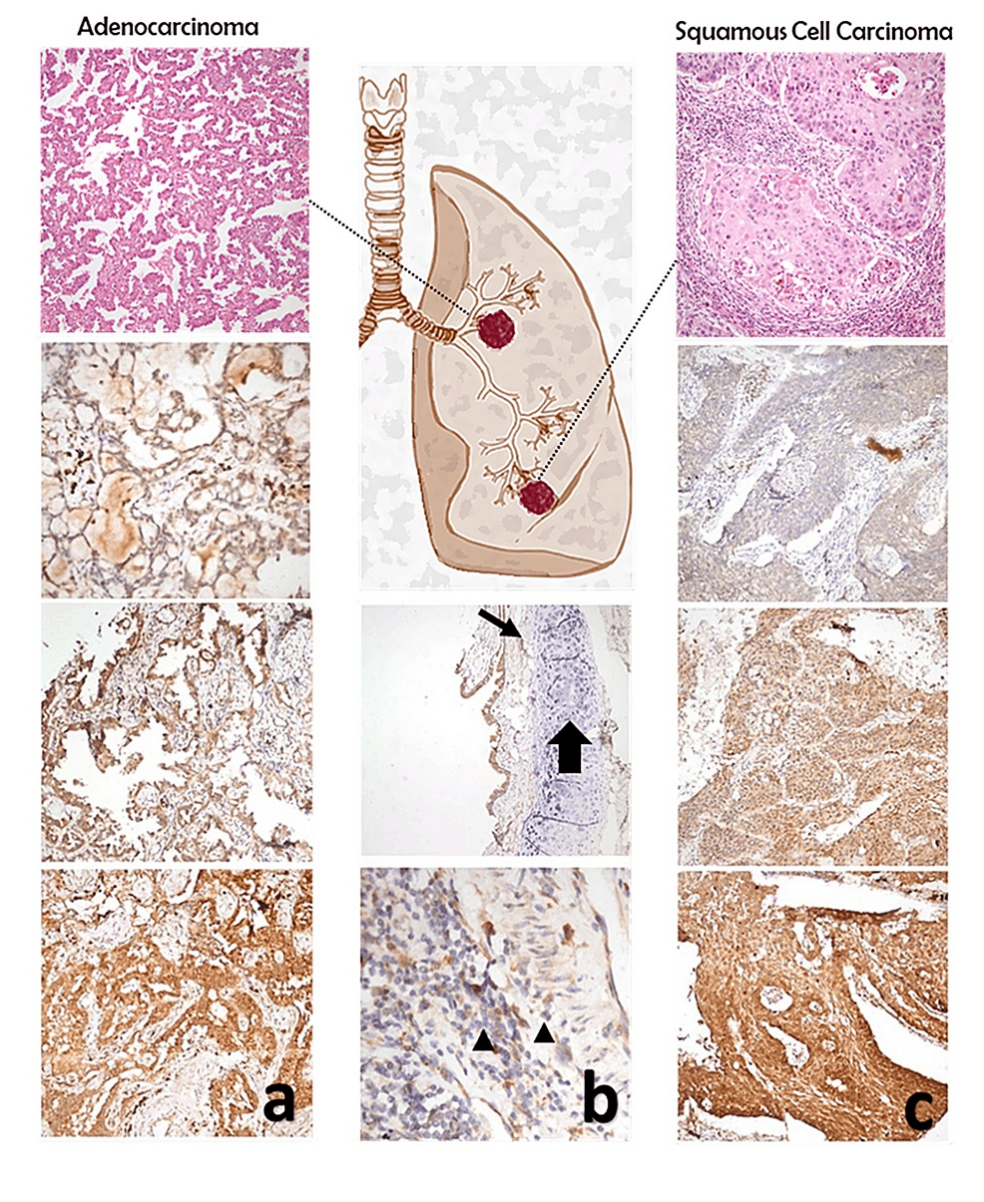
Immunohistochemistry staining of tumor and adjacent non-tumor tissues Representative IHC staining for the KIAA1522 protein in NSCLC tumors and adjacent control tissues; from up to downward, intensity was scored 1, 2, and 3, respectively. (a) IHC staining intensity in adenocarcinoma tissues. (b) IHC staining pattern in normal adjacent lung tissue; thin arrow indicates weak staining in respiratory ciliary epithelia, thick arrow indicates no staining in cartilage tissue, arrowhead indicates no staining in lymphocytes. (c) IHC staining intensity in squamous cell carcinoma tissues. IHC, immunohistochemistry.

Paraffin-embedded tissue specimens of patients were identified, and tissue sampling from paraffin blocks was made by punch biopsy knife with a 4 mm radius. Sampling was made twice for each case, and antigen retrieval was done in an automatized Leica Bond-Max machine, and samples were incubated with primary rabbit polyclonal antibody anti-KIAA1522 (Sigma-Aldrich© HPA032050-100UL). After the dilution process, incubation with anti-rabbit poly-HRP-IgG antibodies (Novolink™ Polymer) was performed. The correct dilution ratio of the stain was decided with control staining in accordance with the given staining patterns at Human Protein Atlas (available from https://www.proteinatlas.org/), and 1:250 was accepted as the optimum ratio. During the assessments of the samples, internal controls were made in each sample; staining of alveolar macrophages was expected to be medium-strong, so accepted as the positive control, and lymphocytes and bronchial cartilage were not supposed to be stained, so accepted as the negative control (Figure 2).

Two pathologists, identified as SO and OA, assessed the sections. One of the pathologists had expertise in pulmonary diseases. They were blinded to the patient outcome, clinical characteristics, previous official reports, and each other's results to ensure unbiased evaluation. In a few instances, minor conflicts were observed, and in such cases, the conflicting samples were reevaluated. However, no significant conflicts were encountered. 

With the percentage and intensity of tumor cell staining, the h score was determined. The percentage of tumor cell staining was categorized into the following scores: 0 (<5%), 1 (6-25%), 2 (26-50%), 3 (51-75%), and 4 (76-100%). The intensity of staining was evaluated at 100x magnification and assigned scores ranging from 0 to 3 (Figure 2). To calculate the final h score for each sample, the average intensity score was multiplied by the score corresponding to the percentage of positive cells. The optimal cutoff value for distinguishing between overexpression and low expression was determined using the maxstat package which employs maximally selected rank statistics with various p-value approximations. Additionally, the suggestions of the two pathologists involved in the study were taken into consideration. Values above the determined cutoff were considered indicative of overexpression, while values below the cutoff were categorized as low expression.

Statistical analysis

The continuous variables were presented by median/mean values and interquartile range/standard deviation, while categorical ones were presented by percentages. The chi-squared test (χ^2^ test) or Fisher's exact test was used for categorical variables, and Student's t-test or the Mann-Whitney U test for continuous variables according to the normality pattern. h score, calculated for the KIAA1522 expression profile and clinicopathological data, was compared. Conventional statistical methods were performed by using various packages in the R 4.2.2 programming language.

Survival analyses utilized multivariate Cox proportional hazards models. Orange 3.34.0 Software (University of Ljubljana, Slovenia) facilitated feature ranking, model development, performance evaluation, and Kaplan Meier graphics generation for PFS and OS in the cohort. Two key metrics were measured: a prognostic index constructed via the linear predictor score, assessed with the receiver operating characteristic (ROC) curve; and the concordance index (Harrel's c-index) indicating event prediction concordance [15]. A c-index above 0.7 generally suggests a good model. Stratified tenfold cross-validation gauged predictive capacity for future events. Kaplan-Meier plots illustrated KIAA1522 expression impact on PFS and OS for non-metastatic patients and the full cohort. Survival rates between distinct expression profiles were compared using the log-rank test. All p-values presented in this study were two-sided and considered significant if below 0.05.

## Results

Baseline characteristics

Samples and data of 98 patients who met our inclusion criteria were studied, including 54 patients with adenocarcinoma (ADC) and 44 with squamous cell carcinoma (SCC). Table 1 shows the clinical characteristics of NSCLC patients grouped by tumor histology (ADC and SCC). The mean age was 60.46 ± 8.81 years, and there was no statistically significant difference between the two groups (t-test 0.07). Male patients seemed to diagnose nearly four times higher than females (80.6% vs. 19.4%). To be a former and active smoker was more likely to be associated with SCC (p = 0.003). Known chronic obstructive pulmonary disease (COPD) history (p = 0.02) and hemoptysis at diagnosis (p = 0.057) were more likely to have SCC. Having metastasis at diagnosis was significantly higher in ADC (Table 2, p = 0.024). As expected, the location of the primary tumor greatly differed between the two groups (<0.001).

**Table 1:** Baseline clinical characteristics by histologic type of 98 patients who underwent surgery for ADC (54 patients) or lung SCC (44 patients) from 2010 to 2015. COPD, chronic obstructive pulmonary disease; CAD, coronary artery disease; CHF, congestive heart failure; CKD, chronic kidney disease; CCl, Charlson comorbidity index; ECOG, Eastern Cooperative Oncology Group Scale; ADC, adenocarcinoma; SCC, squamous cell carcinoma.

Parameter	Total N = 98	Adenocarcinoma N = 54	Squamous cell carcinoma N = 44	p-Value
Age (mean ± SD)	60.46 ± 8.81	59.04 ± 9.84	62.21 ± 8.7	0.07
Sex				
Female	19 (19.4%)	13 (24.1%)	6 (13.6%)	0.0194
Male	79 (80.6%)	41 (75.9%)	38 (86.4%)	
Smoking status				
Active smoker	44 (44.9%)	23 (42.6%)	21 (47.7%)	0.003
Ex-smoker	29 (29.6%)	10 (18.5%)	19 (43.2%)	
Non-smoker	16 (16.3%)	14 (25.9%)	2 (4.5%)	
Symptoms before diagnosis				
Cough	38 (39.6%)	19 (36.5%)	19 (43.2%)	0.507
Dyspnea	17 (17.7%)	11 (21.2%)	6 (13.6%)	0.336
Chest pain	11 (11.5%)	7 (13.5%)	4 (9.1%)	0.541
Back pain	6 (6.3%)	2 (3.8%)	4 (9.1%)	0.29
Hemoptysis	11 (11.5%)	3 (5.8%)	8 (18.2%)	0.057
Fatigue	13 (14.8%)	5 (11.4%)	8 (18.2%)	0.36
Weight loss	13 (13.5%)	7 (13.5%)	6 (13.6%)	0.98
Comorbidity				
Hypertension	35 (35.7%)	15 (27.8%)	20 (45.5%)	0.069
Diabetes mellitus	17 (17.4%)	8 (14.8%)	9 (20.4%)	0.68
COPD	33 (33.7%)	12 (24.1%)	20 (45.5%)	0.02
CAD	14 (13.3%)	9 (16.7%)	4 (9%)	0.405
CHF	5 (5.1%)	2 (3.7%)	3 (6.8%)	0.48
CKD	4 (4.1%)	3 (5.6%)	1 (2.3%)	0.605
CCl ≥4	23 (24%)	13 (25%)	10 (22.7%)	0.79
ECOG >1	16 (16.3%)	9 (16.7%)	7 (15.9%)	0.92

**Table 2:** Baseline tumor characteristics by histologic type of 98 patients who underwent surgery for ADC (54 patients) or lung SCC (44 patients) from 2010 to 2015 ADC, adenocarcinoma; SCC, squamous cell carcinoma.

Parameter	Total (%)	Adenocarcinoma	Squamous cell carcinoma	p-Value
Tumor size >3 cm	59 (61.5%)	31 (61.5%)	27 (61.4%)	0.98
Lymph node involvement	47 (48%)	26 (48.1%)	21 (47.7%)	0.96
Mediastinal node involvement	23 (23.5%)	18 (33.3%)	5 (11.4%)	0.01
Visceral pleural involvement	26 (30.2%)	12 (26.1%)	14 (35%)	0.37
Surgery margin positivity	5 (5.7%)	1 (2.2%)	4 (9.8%)	0.12
Metastasis				
No metastasis	82 (83.7%)	40 (74.1%)	42 (51.2%)	0.024
Pleural effusion or contralateral lung involvement	3 (3.1%)	3 (5.6%)	0 (0.0%)	
Single extrathoracic metastasis	6 (6.1%)	6 (11.1%)	0 (0.0%)	
Multiple extrathoracic metastases	7 (7.1%)	5 (9.3%)	2 (4.5%)	
TNM stages				
Stage I-II	60 (61.2%)	31 (57.4%)	29 (65.9%)	0.39
Stage III-IV	38 (38.8%)	23 (42.6%)	15 (34.1%)	
Location				
Superior sulcus tumor	7 (7.1%)	4 (7.4%)	3 (6.8%)	<0.001
Peripheral	44 (44.9%)	39 (72.2%)	5 (11.4%)	
Central	47 (48.0%)	11 (20.4%)	36 (81.8%)	
Diagnostic procedure				
Tru-cut biopsy	25 (25.5%)	16 (29.6%)	9 (20.5%)	<0.001
Forceps biopsy	22 (22.4%)	3 (5.6%)	19 (43.2%)	
Surgical excision	37 (37.8%)	26 (48.1%)	11 (25.0%)	
Fine-needle	14 (14.3%)	9 (16.7%)	5 (11.4%)	
Pathological sample				
Lobectomy	68 (69.4%)	38 (70.4%)	30 (68.2%)	0.12
Pneumoctomy	17 (17.3%)	6 (11.1%)	11 (25%)	
Wedge resection	2 (2%)	2 (3.7%)	0 (0%)	
Metastasectomy	11 (11.2%)	8 (14.8%)	3 (6.8%)	

Relationship between KIAA1522 and clinicopathologic data

All specimens showed KIAA1522 staining in tumor cells for both subtypes. Regarding the scoring model suggested by two pathologists involved in our study and the literature, h scores between 9 and 12 were nominated as high expression, and all other results were regarded as low expression. Non-tumoral tissue displayed consistent weak staining as internal controls. Alveolar macrophages exhibited strong staining, while cartilage and lymphocytes remained unstained. Normal respiratory epithelia displayed weak staining, aligning with Human Protein Atlas patterns. H scores did not differ between ADC and SCC (Table 3). No significant links emerged between KIAA1522 expression and age, sex, smoking status, comorbidities, tumor size, lymphatic spread, or TNM stages. Yet, progressive disease is linked to high expression (p = 0.046; Table 4).

**Table 3:** Immunohistochemical characteristics of tumor tissues

Parameter	Total (%)	Adenocarcinoma	Squamous cell carcinoma	p-Value
Immunohistochemistry				
TTF1	48 (53.3%)	46 (86.8%)	2 (5.4%)	<0.001
P63	34 (73.9%)	2 (7.1%)	32 (69.5%)	<0.001
CK7	38 (71.7%)	35 (97.2%)	3 (5.7%)	<0.001
CK5-6	20 (77%)	3 (44.6%)	17 (88.8%)	0.043
Mucin	15 (50%)	15 (92.2%)	0 (0%)	<0.001
KIAA1522 antibody staining				
Intensity score				
1	9 (9.2%)	5 (9.3%)	4 (4.1%)	0.95
2	35 (35.7%)	20 (37%)	15 (34.1%)	
3	54 (55.1%)	29 (53.7%)	25 (56.8%)	
Percentage				
0-25%	1 (1.0%)	1 (1.9%)	0 (0%)	0.055
26-50%	5 (5.1%)	1 (1.9%)	4 (91%)	
51-75%	9 (9.2%)	8 (14.8%)	1 (2.3%)	
76-100%	83 (84.7%)	44 (44.9%)	39 (39.8%)	
h Score				
Low expression	44 (44.9%)	25 (46.3%)	19 (43.2%)	0.75
High expression	54 (55.1%)	29 (29.6%)	25 (56.8%)	

**Table 4:** Baseline characteristics by KIAA1522 expression groups of 98 patients who underwent non-small-cell lung cancer resection for ADC (54 patients) or lung SCC (44 patients) from 2010 to 2015 TNM, tumor, node, metastasis; ADC, adenocarcinoma; SCC, squamous cell carcinoma.

	KIAA1522 expression groups		
	Low expression	Overexpression	p-Value
Age	61.13 ± 7.4	59.92 ± 9.8	0.504
Sex			
Male	37 (84.1%)	42 (77.8%)	0.45
Female	7 (15.9%)	12 (22.2%)	
Tumor histology			
ADC	25 (58.9%)	29 (46.3%)	0.76
SCC	19 (43.2%)	25 (46.3%)	
Tumor size			
≤3 cm	18 (40.9%)	19 (36.5%)	0.68
>3 cm	26 (59.1%)	33 (63.5%)	
Lymph node involvement			
No	28 (63.6%)	23 (42.6%)	0.038
Yes	16 (36.4%)	31 (57.4%)	
Mediastinal node involvement			
No	39 (39.8%)	36 (66.7%)	0.011
Yes	5 (11.4%)	18 (33.3%)	
Visceral pleural involvement			
No	31 (72.1%)	29 (67.4%)	0.64
Yes	12 (27.9%)	14 (32.6%)	
Surgery margin positivity			
No	41 (95.3%)	41 (95.3%)	0.98
Yes	2 (2.3%)	3 (6.8%)	
Metastasis			
No	82 (83.7%)	40 (74.1%)	0.44
Yes	3 (3.1%)	3 (5.6%)	
TNM 8 Stages			
I	19 (43.2%)	19 (35.2%)	0.45
II	13 (29.5%)	12 (22.2%)	
III	7 (15.9%)	12 (22.2%)	
IV	5 (11.4%)	11 (20.4%)	
Progression			
No	33 (76.7%)	31 (57.4%)	0.046
Yes	10 (23.3%)	23 (42.6%)	
Relapse			
No	32 (82.1%)	29 (67.4%)	0.13
Yes	7 (17.9%)	14 (32.6%)	

Hazard models and survival analysis

**Figure 3:**
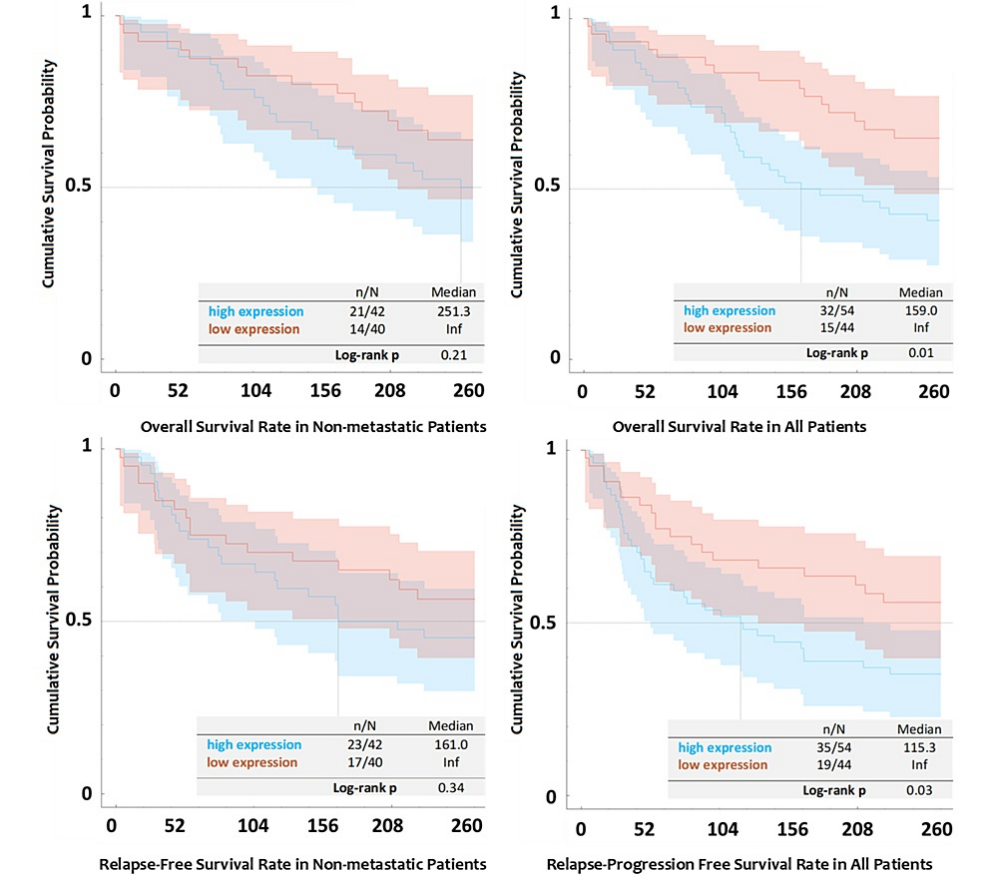
Kaplan-Meier graphics Kaplan-Meier curves showing the association between expression of KIAA1522 and patients' PFS and OS in non-metastatic patients and the entire cohort (p values are shown in the graph, calculated through ‘log-rank test’). PFS, progression-free survival; OS, overall survival.

To evaluate the impact of the high KIAA1522 expression on survival parameters (OS and PFS), multivariate analyses were conducted, considering clinicopathological characteristics along with the KIAA1522 expression profile (refer to Tables 5, 6). The prognostic significance of KIAA1522 was further investigated using the log-rank test, and the differences between expression profiles were illustrated in Kaplan-Meier graphics (Figure 3). Factors that could potentially influence OS and PFS rates in patients with NSCLC were included in the log-likelihood ratio test, and the five highest-ranking features were incorporated into Cox proportional hazard models.

**Table 5:** Cox proportional hazard models for overall survival in non-metastatic and all patients. TNM, tumor, node, metastasis; ECOG, Eastern Cooperative Oncology Group; AUROC, area under the curve of the receptor operating characteristics value for linear predictor score in each model; c-index, the concordance index for Cox regression.

Cox proportional hazard models for overall survival							
		Non-metastatic patients			All patients		
	Wald	HR	95% CI	Wald		HR	95% CI
Age	22.360	1.128	1.073-1.18	14.786		1.062	1.03-1.09
TNM stages	2.916	1.45	0.96-2.24	9.241		1.560	1.12-3.01
Visceral pleural involvement	3.062	1.9	0.95-3.8	2.404		1.636	0.8-3.04
ECOG performance score	2.601	1.64	0.9-3	5.872		1.840	1.17-2.07
High KIAA1522 expression	1.848	1.102	0.95-1.26	4.564		1.149	1.012-1.3
	AUROC value: 0.86, c-index: 0.72			AUROC value: 0.87, c-index: 0.74			

**Table 6:** Cox proportional hazard model for progression-relapse-free survival in non-metastatic and all patients. TNM, tumor, node, metastasis; ECOG, Eastern Cooperative Oncology Group; AUROC, area under the curve of the receptor operating characteristics value for linear predictor score in each model; c-index, the concordance index for Cox regression.

Cox proportional hazard models for progression-relapse-free survival							
		Non-metastatic patients			All patients		
	Wald	HR	95% CI	Wald		HR	95% CI
Age	15.7	1.1	1.053-1.16	3.280		1.028	0.99-1.06
TNM stages	5.31	1.72	1.085-2.73	15.980		1.809	1.3-2.4
Visceral pleural involvement	3.01	1.9	0.92-4	2.153		1.577	0.8-2.89
ECOG performance score	2.9	1.75	0.92-3.3	6		1.891	1.13-3.14
High KIAA1522 expression	1.9	1.1	0.96-1.27	4.146		1.138	1.005-1.28
	AUROC value = 0.84, c-index= 0.71			AUROC value = 0.83, c-index = 0.72			

The models built on KIAA1522 expression (high vs. low), age, visceral pleural involvement, ECOG performance score, and TNM stages are presented in Tables 5, 6. High KIAA1522 expression, advanced TNM stages, poorer ECOG performance score, and older age were identified as independent prognostic factors for the risk of death in NSCLC patients. However, in non-metastatic patients, the association between KIAA1522 expression and mortality approached but did not reach a significant level.

Another proposed model focused on PFS is presented in Table 6, and in the model for non-metastatic patients, the KIAA1522 expression profile did not emerge as a significant independent predictor for relapse-free survival. However, when metastatic patients were included in the model, the KIAA1522 expression profile significantly predicted the time to relapse or disease progression. Similarly, the assessment of KIAA1522's impact on OS and PFS using the log-rank test was significant for the entire cohort, but not significant when metastatic patients were excluded from the analysis (Figure 3).

## Discussion

Our study discerned three key factors significantly influencing overall survival in patients: a high ECOG performance score, advanced TNM staging, and increased age. Among these, advanced age is already recognized as a prognostic indicator that can potentially affect disease progression and amplify the adverse effects of treatments [16,17]. Moreover, the performance status, especially for patients aged 65 and above at the point of diagnosis, has always presented challenges in the management of NSCLC [18-20]. However, it is notable that younger patients with robust performance scores, even those at relatively early stages or with oligometastatic disease, are not exempt from potential relapses, treatment side effects, or resistance to standard treatment protocols. Predicting these adverse trajectories is vital, as it empowers treating physicians to customize therapy plans for those demonstrating poor prognostic indicators. In this context, reliable and validated biomarkers can prove invaluable.

Recent studies have highlighted the potential of KIAA1522 in detecting tumoral tissues and influencing the survival rate. Specifically in the context of NSCLC, KIAA1522 has shown promise in early diagnostic processes [11]. Beyond its diagnostic prowess, KIAA1522 has carved its niche as a potent biomarker for predicting patient survival and gauging the chemotherapy response [7,12]. An intriguing observation from gene-set enrichment analyses revealed a concerning correlation: elevated KIAA1522 levels and genes demonstrating poor survival outcomes [7]. The connection of KIAA1522 with KRAS activation, a known element in chemotherapy resistance, further accentuates its significance in NSCLC [21]. Observations have shown that by inhibiting the MEK pathway, KIAA1522 expression diminishes, thereby strengthening its ties to the RAS-MEK-ERK pathway.

Delving deeper into cellular pathways, we noted a robust association between KIAA1522 and the TNFα-NFκB signaling mechanism. Evidence suggests that depletion in KIAA1522 aligns with cisplatin-induced cell mortality, postulating its role in therapeutic resistance through enhanced TNFα-NFκB signaling [12]. Given that platinum-based chemotherapy is the standard treatment for NSCLC, this association holds utmost importance. Emerging data posit KIAA1522 as a potential inducer of platinum insensitivity, commonly found in aggressive NSCLC variants, with an active role in tumorigenesis. Additionally, similar to the correlation observed between KIAA1522 and KRAS and MEK, increased ERK activity has been linked to KIAA1522 overexpression and is associated with tumorigenesis and metastasis in esophageal cancer cells [8]. Furthermore, a recent study focusing on a particular circular RNA, known to be highly expressed in NSCLC tissues, demonstrated its promoting effect on NSCLC tumorigenesis by upregulating KIAA1522 expression [22].

While it is unarguably sensible to investigate the association and correlation of explored genes, transcripts, and signaling pathways to understand the biological function of a single protein, in vivo measures will always be confounded by clinical characteristics. Therefore, assessing the clinical impact of a biomarker requires analyzing real-life parameters such as TNM stages, gender, smoking behavior, and age. Biomarkers that can be used as surrogates indicate specific histological, genetic, and epigenetic changes, providing improved risk stratification and anticipated response to different treatment modalities.

Our study has certain strengths and limitations. The study design necessitated the exclusion of patients diagnosed using cytological cell blocks and small biopsy samples. Such exclusions stemmed from ethical concerns as using these samples for research could limit their clinical utility. This inadvertently reduced our population size, potentially masking the effects of overexpression in more niche groups. Another limitation was that we only investigated our samples with IHC whereas using transcriptomics technologies, cell cultures, or animal models would have provided more detailed data on upstream and downstream molecular pathways. However, the study’s strength lies in demonstrating the effect of overexpression on relapse progression and mortality. There was a significant association between prognosis and expression profile, although hazard models did not show the prognostic significance of KIAA1522 overexpression when metastatic patients were excluded. Despite the lack of predictive value in non-metastatic patients, the difference between the two groups was visually evident in Kaplan-Meier graphics, suggesting that the lack of prediction may be primarily due to the small size of the population. Possibly, curative approaches may eliminate the tumor cells, reducing the potential detrimental impact of KIAA1522-overexpressed cells, or KIAA1522 overexpression may exert its aggressiveness in metastatic patients more than in patients who received curative surgery. Further investigations involving a larger, and ideally prospective, sample set would be beneficial for corroborating these findings.

## Conclusions

Our work proposes a model for predicting the disease's course based on the overexpression of a potential biomarker using IHC and other classical influencers of the disease. Although the broader use of genomic and proteomic studies would yield more reliable data, IHC remains a simple, low-cost, and more feasible identification method to show the overexpression of certain proteins. This work will inspire further investigation into other uncharacterized proteins associated with poor survival, potentially leading to a multi-protein approach using genomic technologies and simple IHC to determine recalcitrance and overall prognosis.
